# Influence of Implantable Hearing Aids and Neuroprosthesison Music Perception

**DOI:** 10.1100/2012/404590

**Published:** 2012-05-03

**Authors:** Torsten Rahne, Lars Böhme, Gerrit Götze

**Affiliations:** Department of Otorhinolaryngology, Head and Neck Surgery, University Hospital Halle (Saale), 06120 Halle, Germany

## Abstract

The identification and discrimination of timbre are essential features of music perception. One dominating parameter within the multidimensional timbre space is the spectral shape of complex sounds. As hearing loss interferes with the perception and enjoyment of music, we approach the individual timbre discrimination skills in individuals with severe to profound hearing loss using a cochlear implant (CI) and normal hearing individuals using a bone-anchored hearing aid (Baha). With a recent developed behavioral test relying on synthetically sounds forming a spectral continuum, the timbre difference was changed adaptively to measure the individual just noticeable difference (JND) in a forced-choice paradigm. To explore the differences in timbre perception abilities caused by the hearing mode, the sound stimuli were varied in their fundamental frequency, thus generating different spectra which are not completely covered by a CI or Baha system. The resulting JNDs demonstrate differences in timbre perception between normal hearing individuals, Baha users, and CI users. Beside the physiological reasons, also technical limitations appear as the main contributing factors.

## 1. Introduction

The perception of music is an important feature of everyday life improving life quality and is also a prerequisite to discriminate speech prosody and speakers. In individuals with hearing loss or deafness, the ability to perceive music is reduced or lost [1, 2]. To enable or restore speech and music perception is the main goal of hearing aids and implantable neuroprosthesis.

One prevalent factor for music perception is the ability to perceive and discriminate timbre. However, timbre appears as a psychoacoustical attribute of complex tones comprising all acoustical attributes that are not exclusively assigned to the perception of pitch, loudness, and subjective duration [3, 4]. Thus, timbre is a multidimensional space, which dimensions differently contribute to the psychoacoustical perception of timbre. In normal hearing listeners, recent work identified the spectral shape, the spectral fluctuation, and the rise time as the most dominating parameters of timbre [5, 6]. Further, the intensity fluctuation appears as one parameter characterizing the timbre difference of instruments [7].

Previous work used complex tones recorded by natural instruments or sound generated by a synthesizer as stimuli to measure timbre discrimination [7–9]. As these stimuli differ in more than one timbre dimension, a control of a single parameter is not possible with these stimuli. To overcome this limitation, a behavioral test using synthetically sounds was developed [10] and evaluated [11]. With this test, cross-faded (morphed) tones were generated to change only the spectral shape difference in certain steps by linear interpolation of spectral parameters. Thus, in contrast to previous studies which generated cross-faded continua of instrumental recordings [7, 12], these tones form a timbre continuum differing only in their spectral shape. In a forced-choice paradigm, the timbre difference was changed adaptively to measure the individual just noticeable difference (JND) [13].

To contribute to a more systematically evaluation of the ability to rehabilitate music perception, our study investigates the individual timbre discrimination skills of individuals with severe-to-profound hearing loss using a cochlear implant (CI) and normal hearing individuals using a bone-anchored hearing aid. A cochlear implant which is applicable for adults and children with severe-to-profound deafness transforms the incoming sound signal to a digital signal by a coding strategy, wherewith the auditory nerve fibers are stimulated. Therefore electrical pulses are delivered by electrodes which are spread along the cochlea. Despite in the majority of cases a sufficient speech intelligibility can be enabled or restored by using recent devices, CI users still report an insufficient perception of music which becomes apparent by a reduced discrimination or recognition of melodies [14].

Factors contributing to a reduced music perception in CI users basically are technical parameters of the CI devices. For example, the number of electrodes (12–22) limits the spectral resolution within the cochlea and thus affects the (fundamental) pitch discrimination [15]. Further, complex pitch perception is currently limited by the temporal resolution of the cochlear implant [16]. Also the limited length of the electrode restricts the stimulation of the low-frequency regions of the cochlea and thus influences the music perception [1, 17]. Concerning the multidimensionality of timbre, it has been reported that the spectral shape seems to be one dominating parameter of timbre discrimination in CI users [18].

Patients suffering from moderate hearing loss may benefit from hearing aids. Beside the differentiation between external, semi-implantable, and fully implantable hearing aids, the devices can also be distinguished by the transmission pathway of the sound to the cochlea. So the direct stimulation of the cranial bone overcomes a conductive hearing loss. The most prominent device is the bone conduction hearing aid (Baha) comprising an implantable titanium screw placed to achieve osteointegration, a percutaneous coupling, and an electromechanical processor. With this a vibration of the cochlea fluid is evoked by a vibrator [19, 20]. For indication criteria, please refer to Bosman et al. [21] and Snik et al. [22].

For testing the performance and the individual outcome of the Baha device, a temporary transcutaneous coupling of the device is provided by the Baha test device and the Baha softband (Cochlear Ltd.). With this, the Baha sound processor is connected to a special adapter and pressed onto the patients' skin. The resulting hearing thresholds and the speech development with the Baha softband are almost equal to them achieved with a conventional bone conductor [23, 24]. As the Baha softband is a reversible and noninvasive method of providing bone conduction hearing, it appears as an appropriate method to simulate measures with normal hearing control groups.

A further aspect concerning the ability to discriminate spectral differences is the limited bandwidths of the electrical devices (Baha, CI). Thus, low-frequency and high-frequency portions of the respective stimulus spectrum are not transmitted by the hearing devices. However, recent investigations exploring the audibility of sound within a frequency spectrum from 500 to 4000 Hz [25] or the audibility of the long-term average speech spectrum [26] found no relevant perceptive deficiency. Nevertheless, the spectrum of music exceeds this frequency range. Thus a systematically investigation of timbre perception would be an appropriated step to investigate the music perception skills of Baha and CI users.

In this study, we investigate the individual spectral shape JND for complex tones in cochlear implant users compared to normal hearing listeners using a Baha or not. To explore the differences in timbre perception abilities caused by the hearing mode, the sound stimuli were varied in their fundamental frequency, thus generating different spectra which are not completely covered by a CI or Baha system.

## 2. Methods

### 2.1. Participants

Twelve normal hearing adults (six females, six males) between the ages of 21–71 years participated in the study. All participants passed a hearing screening (pure tone thresholds of 10 dB HL or better from 250 to 4000 Hz in both ears) and had no reported history of hearing or neurological problems. For the second part of the experiment, these patients were fitted with a bone-anchored hearing aid (Baha Intenso, Cochlear Ltd., Australia). The vibrator was connected to the right mastoid using the Baha softband (Cochlear Ltd., Australia).

Ten adults (nine females, one male; 32–72 years of age) using a unilateral Nucleus CI (models CI24M, CI24R, CI24RE, or CI512; fitted with an ESPrit 3G, Freedom SP, or CP810 sound processor) or an unilateral MED-El CI (model SONATA TI 100; fitted with an OPUS 2 sound processor) with no residual hearing (pure tone threshold >80 dB HL from 250 to 4000 Hz) on the other side were enrolled in the study. Subject demographics are shown in Table 1.

All participants were assessed for their musical experience. They gave informed consent after the procedures were explained to them, in accordance with the ethical guidelines of the Martin-Luther-University Halle-Wittenberg, where the study was conducted. The procedures conform to the Code of Ethics of the World Medical Association (Declaration of Helsinki).

### 2.2. Stimuli

**Figure 1:**
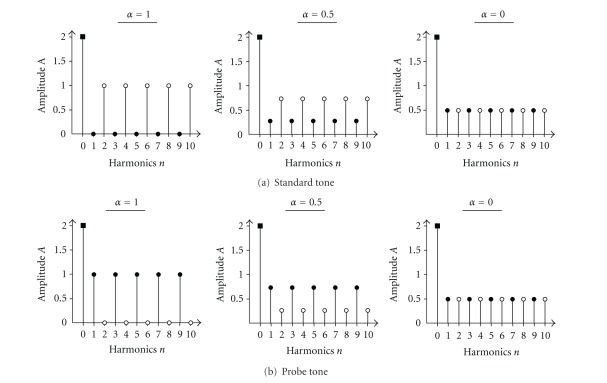
Amplitude spectrums of the standard (upper row) and probe stimuli waveforms (lower row). The amplitude of the fundamental frequency (*n* = 0) remains constant, while the amplitudes *A* of the even (white circles) and odd harmonics (black circles) were cross-faded by the parameter *α*. Applying no cross-fading (*α* = 1), the amplitudes are maximal different which results in a maximal timbre difference. The amplitudes of all harmonics are equal at maximal cross-fading (*α* = 0) which results in stimuli with no timbre difference.

The stimuli were calculated with the MATLAB software as complex waveforms with a fundamental frequency *f*_0_ of 65.5, 131, 262, 524, or 1048 Hz. With each of the five fundamental frequencies, ten harmonics were added with 50% of the fundamental frequency amplitude, thus covering a frequency range from 2 × *f*_0_ to 11 × *f*_0_. For each of the five fundamental frequencies, a standard and a probe stimulus was calculated. At maximal spectral shape difference, the amplitudes of the odd harmonics were set to zero for the standard stimuli and the amplitudes of the even harmonics were set to zero for the probe stimuli. Thus, a combshaped amplitude spectrum occurred for the different tone sets (see Figure 1). The resulting complex waveforms were calculated as superposition of sine waves without a phase shift, consisting of the fundamental frequency and the harmonics. 

**Figure 2:**
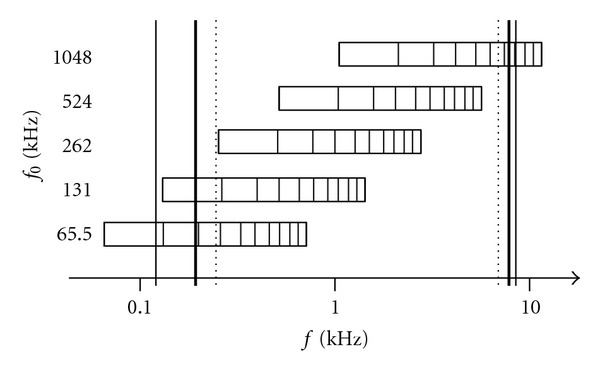
Amplitude spectrums of the *f*_0_ = 65.6 Hz, 131 Hz, 262 Hz, 524 Hz, and 1048 Hz stimuli with ten harmonics with respect to the maximal (thin line) and minimal frequency range (thick line) of the CI speech processors as well as the frequency spectrum of the Baha Intenso device (dotted line). The spectra of the *f*_0_ = 65.6 Hz, *f*_0_ = 131 Hz, and *f*_0_ = 1048 Hz tones are not fully covered by the transmission spectra of the CI and the Baha Intenso.

The frequency spectrum of the most stimuli covers the frequency range of a cochlear implant or a Baha Intenso system (see Figure 2). The spectra of the waveforms with fundamental frequencies of *f*_0_ = 65.5 Hz and *f*_0_ = 131 Hz start with frequencies below the low cutoff frequency of the hearing systems. Only the spectrum of the *f*_0_ = 1048 Hz stimulus exceeds the high cutoff frequency of the hearing systems.

The stimulus duration was 700 ms containing 75 ms of rise and fall time periods. The amplitudes of the resulting waveforms were scaled to achieve a sound pressure level of 65 dB. To assure a constant loudness of all used stimuli, the sound pressure level of the stimuli with a reduced spectral difference was measured exemplary for spectral differences of *α* = 0.1 and *α* = 0.5. Also with these spectral differences, the sound pressure level was 65 dB. Thus, loudness (probably) could not be used as a cue for the discrimination of the sounds. The sound pressure level of the stimuli was calibrated using a sound level meter (type 2235, B&K). An external soundcard (DMX 6 Fire, Terratec) with a resolution of 24 Bits was used as D/A converter and connected to the stimulation computer. A Power amplifier (POA-800, Denon) and a free field studio loudspeaker (Reveal 6, Tannoy) with a distance of 1 m in front of the listener were used to present the stimuli in free field conditions in an acoustically shielded room.

For the Baha users, the sound signals were delivered directly to the line input of the Baha processor. The opposite ear canal was closed with a wax ear plug (Ohropax, Germany) to avoid an influence of the sound signal emitted by the Baha on the psychoacoustical task. The program switch of the Baha Intenso was set to “external”, and the gain control was set to its middle position which provided a linear I/O function of the sound intensity level up to 60 dB HL. As the Baha modifies the sound intensity level, the calibration of the sound intensity was done by a psychoacoustical procedure before running the experiment. Therefore, the participants were simultaneously using a headphone and the Baha. Alternating to the sound delivered electrically to the external audio port of the Baha Intenso a calibrated signal (65 dB HL) was presented by the headphone. Comparing the perceived sound intensity levels, the volume switch of the Baha was adjusted until the perceived sound level intensities were rated as equal. This resulted in a volume value of “2” for every subject. To avoid low-frequency artifacts but ensure the transmission of the used stimulus frequencies, the tone control of the Baha Intenso was adjusted to the middle position.

### 2.3. Experimental Setup and Procedure

All participants performed a psychoacoustically test protocol. For each of the five fundamental frequencies, a timbre discrimination test was performed. The resulting five conditions were run in a quasirandomized order. All normal hearing participants repeated this protocol using the bone-anchored hearing aid. In this test, the conditions were again pseudorandomized.

In every condition, the pairs of standard and deviant stimuli with an equal fundamental frequency were presented in a 3-alternative forced choice (AFC) paradigm. In every trial, two standard stimuli and one probe stimulus were presented subsequently in a randomized order. The participants' task was to listen carefully to the three subsequent stimuli. They were informed that one of three stimuli will differ in timbre. Thereafter the participants were asked to decide which one of the preceding stimuli was different and to press the respective button. The cross-fading parameter *α*(0 ⋯ 1), representing the amplitude difference between the odd and even harmonics for both the standard and the probe stimuli, was initially set to *α* = 1. At this maximal superthreshold spectral shape difference, the amplitude of the even harmonics of the standard was 1 and the amplitude of the odd harmonics was zero. At maximal cross-fading (*α* = 0), the stimuli were equal in their spectral shape with an amplitude of the harmonics of 0.5 (see Figure 1). The spectral shape difference was modified adaptively in a 1-up 2-down paradigm converging into the 70.7% point of the psychometric function. After two correct responses, the spectral shape difference of the consequent trial was reduced; after one false response the difference was increased. At passing a minimum of the response function, additionally the step size of the cross-fading change was reduced. Starting with a step size of 0.5, after 8 minima of the response function, the step size reached a value of 0.002. With this precision, the spectral shape JND was calculated as mean *α* of the last 10 responses. For more details regarding the calculation of the response function, please refer to Rahne et al. [10]. 

### 2.4. Data Analysis

The cross-fading parameter *α* represents a linear measure of the amplitude differences of the odd and even harmonics between the standard and the probe stimuli. As the JND refers to the level of a sensory stimulus, the individual spectral shape JND was calculated by logarithmizing the cross-fading parameter *α* to refer the JND to the sound pressure level difference between the respective harmonics.

For every stimulus condition, the individual JNDs were compared by independent *t*-tests between the normal hearing participants and the CI users. For all participants, a two-way analysis of variances (ANOVA) with factors of hearing mode (normal hearing, Baha, CI) and condition (*f*_0_ = 65.5 Hz, 131 Hz, 262 Hz, 524 Hz, and  1048 Hz) was performed on the resulting cross-fading parameter *α* (JND). Resulting effects were specified post hoc with *t*-tests and Bonferroni-corrected probabilities.

## 3. Results

**Figure 3:**
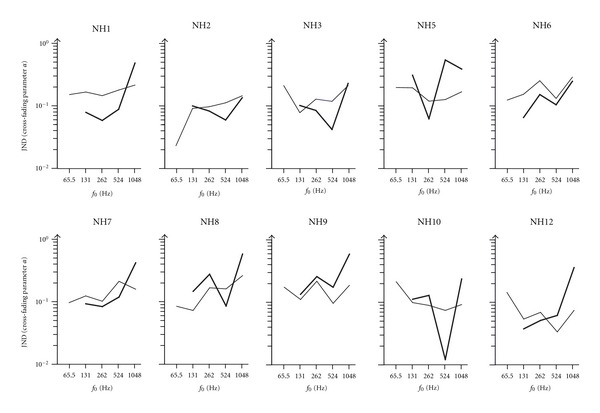
Individual spectral shape JND for the stimulus conditions of *f*_0_ = 65.6 Hz, 131 Hz, 262 Hz, 524 Hz, and  1048 Hz as revealed by the normal hearing participants using a Baha (thick line) or not (thin line). Using the Baha, the resulting JND is poorer in the *f*_0_ = 1048 Hz condition and no JND could be measured in the *f*_0_ = 65.6 Hz condition.

**Figure 4:**
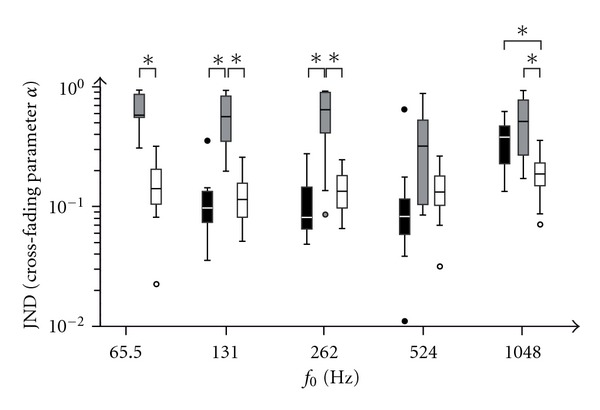
Distribution of the individual spectral shape JND for the normal hearing participants using a Baha (black) or not (white) as well as the CI users (grey) as boxplots for the stimulus conditions of *f*_0_ = 65.6 Hz, 131 Hz, 262 Hz, 524 Hz, and  1048 Hz. Significant differences are marked (*P < 0.05). The globally poorer JND in the CI users and the poorer JND in the Baha users compared to the normal hearing individuals in the *f*_0_ = 1048 Hz condition is obvious.

All normal hearing participants completed the timbre discrimination test. Figure 3 displays the individual spectral shape JND with the normal hearing participants for both hearing modes (Baha, normal hearing) and stimulus conditions (fundamental frequencies). The Baha users could not complete the *f*_0_ = 65.5 Hz condition. Here, no timbre differences were perceived at all. Two normal hearing participants (NH4, NH11) did not agree to the Baha testing. All except of one CI user (CI6) who could not complete the *f*_0_ = 65.5 Hz condition completed the timbre discrimination test.  Figure 4 displays the individual spectral shape JND as boxplots for the different stimulus conditions and groups of hearing modes. For the cross-fading parameter *α*, values between 0.012 and 0.938 were measured. Thus, no ceiling effect occurred for all the CI users and the normal hearing participants if using the Baha or not.

All participants reported none ore school-level musical education. Thus the influence of musical experience was neglected and a two-way ANOVA (hearing mode: normal hearing, Baha, CI × condition: *f*_0_ = 65.5 Hz, 131 Hz, 262 Hz, 524 Hz, and  1048 Hz) was performed on the resulting cross-fading parameter *α* (JND).The results show that the main effect of the hearing mode significantly affected the individual JND, *F*(2, 135) = 58.67, *P* < 0.001. Bonferroni post hoc tests revealed that the JND in cochlear implant users was significantly poorer compared to normal hearing participants and the Baha users (both *P*s < 0.05).

The main effect of the condition was also significant, indicating that the individual JND was elicited differently by the different stimuli spectra (*F*(4, 135) = 6.58,  *P* < 0.001). Bonferroni post hoc tests revealed that the JND with complex tones based on a fundamental frequency of *f*_0_ = 1048 Hz was significantly poorer compared to complex tones with *f*_0_ = 524 Hz, *f*_0_ = 262 Hz, and *f*_0_ = 131 Hz. Further, the JND with *f*_0_ = 65.5 Hz was poorer than the JND with the *f*_0_ = 524 Hz tones (all *P*s < 0.05).

The condition × hearing mode interaction was significant, indicating that the influence of the stimuli spectra was different between the hearing modes (*F*(7, 135) = 2.87,  *P* < 0.01). Post hoc comparisons revealed a better JND with *f*_0_ = 65.5 Hz, *f*_0_ = 131 Hz, *f*_0_ = 262 Hz, and *f*_0_ = 1048 Hz conditions in normal hearing participants compared to the CI users. The JND in Baha users was significantly better than in CI users only for fundamental frequencies of *f*_0_ = 131 Hz and *f*_0_ = 262 Hz. Also in the *f*_0_ = 1048 Hz condition the JND was significantly better in normal hearing participants compared to using a Baha. No significant different JNDs were found between the normal hearing participants and the CI users in the *f*_0_ = 524 Hz condition, between normal hearing participants and Baha users in the *f*_0_ = 131 Hz, *f*_0_ = 262 Hz, and *f*_0_ = 524 Hz conditions, and between Baha users and cochlear implant users in the *f*_0_ = 524 Hz and *f*_0_ = 1048 Hz conditions.

## 4. Discussion

The results show distributions of individual JNDs for the conditions and hearings modes. That is, with the used stimuli timbre discrimination was possible for all participants. As no ceiling occurred, the used psychoacoustical test appears as appropriated test instrument.

However, the results differed between the normal hearing participants if using a Baha or not and the CI users. On average, both the normal hearing participants and the Baha users revealed a better spectral shape JND over all conditions than the CI users did. That is, the ability to discriminate spectral differences is significantly reduced in the CI users. However, there is an overlap of the JND distributions of the CI users and the normal hearing group with all fundamental frequencies (see Figure 4). Consequently, the spectral shape JND of CI users can in principle achieve the level of the normal hearing individuals.

Considering the different stimulus conditions, the response following the *f*_0_ = 524 stimulus elicited significantly better JNDs than the other stimulus conditions did. This might be due to the limited frequency range of the CI which excludes some parts of the stimuli from the signal processing (i.e., the fundamental frequency and the first harmonic of the *f*_0_ = 65.5 Hz stimulus, the fundamental frequency of the *f*_0_ = 131 Hz stimulus, and the last four harmonics of the *f*_0_ = 1048 Hz stimulus; see Figure 2). Thus, a limited amount of information has been available with these stimuli to discriminate the spectral shape. The JND with the *f*_0_ = 524 Hz stimulus which is fully caught by the CI signal processing is in some CI users comparable to the JND of the normal hearing group. Consequently, the limited discrimination skills of spectral differences in CI users would occur mostly in cases when amplitude differences in harmonics occur below and above the frequency transmission range of the CI system.

However, also the *f*_0_ = 262 Hz stimuli which components are completely in the signal processing bandwidth of the CI system have induced a poorer JND in comparison to the *f*_0_ = 524 Hz stimulus. This might be due to physiological restrictions in the CI users. With the low-frequency stimuli up to *f*_0_ = 262 Hz, the fundamental and a part of the harmonics have a pitch which would be physiologically processed in the apical area of the cochlea which is not reached by the CI electrodes. Thus, the respective pitch information has to be delivered only by the temporal information which might have induced the poorer spectral shape JND in the CI users.

The observed interindividual differences of the spectral shape JND in the CI users group might be caused by the different absolute electrode positions in the cochlea of the participants. A varying distance between the electrodes and the modiolus affects the channel interactions and thus the frequency resolution of the CI system. Thus, timbre discrimination might be affected, too. Beside technical and physiological limitations also individual prerequisites as the level of audioverbal rehabilitation, the duration of CI use, and the duration of deafness obviously affect the timbre discrimination skills as well.

In normal hearing individuals, the JND distributions are comparable between using a Baha or not. Only with the low-frequency and high-frequency stimuli the JND if using a Baha is poorer (*f*_0_ = 1048 Hz) or not measurable *f*_0_ = 65.5 Hz as compared to the conservative listening without the Baha. In the high-frequency condition (*f*_0_ = 1048 Hz), the poorer JNDs might have been induced in the Baha users as the spectrum of the *f*_0_ = 1048 Hz stimulus exceeds the high cutoff frequency of the Baha system (see Figure 2). In this condition, the last five harmonics were not transmittable by the Baha system. The information of the resulting five harmonics within the frequency transmission range was obviously not enough to achieve timbre discrimination JND comparable to the normal hearing participants without a Baha. In the *f*_0_ = 65.5 Hz, condition the first harmonic has a frequency below the low-frequency cutoff frequency of the Baha processor. Obviously this harmonic is of significant interest for the discrimination of timbre.

In the *f*_0_ = 131 Hz, *f*_0_ = 262 Hz, and *f*_0_ = 524 Hz conditions, no significant difference was observed between the JNDs in normal hearing participants if using a Baha or not. Here, the sound transmission of the Baha obviously completely recovers the spectral shape as the relevant features of the stimuli to discriminate timbre. However, the stimulation was different between the Baha hearing mode and the normal hearing mode as the Baha was connected only monaural, whereas the stimuli were delivered binaurally to the participants in free-field conditions. Comparing the monaural Baha stimulation and the binaural free-field stimulation, no significant different JNDs were observed. Thus, an improved pitch perception by binaural stimulation [27, 28] could not be observed comparing the different hearing modes.

## 5. Conclusions

The observed timbre discrimination skills measured as JND by changing the spectral shape of complex tones demonstrate differences in music perception between normal hearing individuals, Baha users, and CI users. Beside the physiological reasons, also technical limitations appear as the main contributing factors. However, all measured individuals could discriminate the spectral shape. Further investigations will extend these findings by changing the spectral fluctuation and the rise time of the tones to assess further dimensions of timbre.

The music perception is limited by the technical parameters of the respective hearing aid or neuroprosthesis. This is important for the creation of individual training tools in the audioverbal therapy for the hearing impaired individuals.

## Figures and Tables

**Table 1:** CI user demographics.

Subject No.	Sex	Ear implanted	Age at implant (years)	Device	Frequency range (Hz)	Sound processor	Strategy	Deafness onset	Musical experience	Duration of CI use (years)
1	F	L	39	CI512	188*⋯*7938	CP810	ACE	prälingual	school	<1
2	F	R	44	CI24RE	188*⋯*7938	Freedom SP	ACE	prälingual	none	4
3	M	R	43	CI24RE	188*⋯*7938	Freedom SP	ACE	postlingual	none	2
4	F	R	39	CI24R	188*⋯*7938	Freedom SP	ACE	perilingual	school	1
5	F	L	72	SONATA TI 100	120*⋯*8568	OPUS 2	FSP	postlingual	school	5
6	F	L	57	CI24M	188*⋯*7938	Freedom SP	ACE	postlingual	none	1
7	F	R	38	SONATA TI 100	150*⋯*7352	OPUS 2	FSP	postlingual	none	1
8	F	L	65	CI24RE	149*⋯*7412	Freedom SP	ACE	postlingual	school	<1
9	F	L	26	CI24R	188*⋯*7938	ESPrit 3G	ACE	postlingual	school	10
10	F	R	52	CI24RE	188*⋯*7938	Freedom SP	ACE	postlingual	school	5
